# Antimicrobial Susceptibility of Enteric Gram Negative Facultative Anaerobe Bacilli in Aerobic versus Anaerobic Conditions

**DOI:** 10.1371/journal.pone.0155599

**Published:** 2016-05-18

**Authors:** Zachary DeMars, Silpak Biswas, Raghavendra G. Amachawadi, David G. Renter, Victoriya V. Volkova

**Affiliations:** 1 Department of Diagnostic Medicine/Pathobiology, Institute of Computational Comparative Medicine, College of Veterinary Medicine, Kansas State University, Manhattan, Kansas, United States of America; 2 Department of Diagnostic Medicine/Pathobiology, College of Veterinary Medicine, Kansas State University, Manhattan, Kansas, United States of America; 3 Center for Outcomes Research and Education, Department of Diagnostic Medicine/Pathobiology, College of Veterinary Medicine, Kansas State University, Manhattan, Kansas, United States of America; Indian Institute of Science, INDIA

## Abstract

Antimicrobial treatments result in the host’s enteric bacteria being exposed to the antimicrobials. Pharmacodynamic models can describe how this exposure affects the enteric bacteria and their antimicrobial resistance. The models utilize measurements of bacterial antimicrobial susceptibility traditionally obtained *in vitro* in aerobic conditions. However, *in vivo* enteric bacteria are exposed to antimicrobials in anaerobic conditions of the lower intestine. Some of enteric bacteria of food animals are potential foodborne pathogens, *e*.*g*., Gram-negative bacilli *Escherichia coli* and *Salmonella enterica*. These are facultative anaerobes; their physiology and growth rates change in anaerobic conditions. We hypothesized that their antimicrobial susceptibility also changes, and evaluated differences in the susceptibility in aerobic *vs*. anaerobic conditions of generic *E*. *coli* and *Salmonella enterica* of diverse serovars isolated from cattle feces. Susceptibility of an isolate was evaluated as its minimum inhibitory concentration (MIC) measured by E-Test^®^ following 24 hours of adaptation to the conditions on Mueller-Hinton agar, and on a more complex tryptic soy agar with 5% sheep blood (BAP) media. We considered all major antimicrobial drug classes used in the U.S. to treat cattle: *β*-lactams (specifically, ampicillin and ceftriaxone E-Test^®^), aminoglycosides (gentamicin and kanamycin), fluoroquinolones (enrofloxacin), classical macrolides (erythromycin), azalides (azithromycin), sulfanomides (sulfamethoxazole/trimethoprim), and tetracyclines (tetracycline). Statistical analyses were conducted for the isolates (*n*≥30) interpreted as susceptible to the antimicrobials based on the clinical breakpoint interpretation for human infection. Bacterial susceptibility to every antimicrobial tested was statistically significantly different in anaerobic *vs*. aerobic conditions on both media, except for no difference in susceptibility to ceftriaxone on BAP agar. A satellite experiment suggested that during first days in anaerobic conditions the susceptibility changes with time. The results demonstrate that assessing effects of antimicrobial treatments on resistance in the host’s enteric bacteria that are Gram negative facultative Anaerobe Bacilli requires data on the bacterial antimicrobial susceptibility in the conditions resembling those in the intestine.

## Introduction

The spread of antimicrobial resistance (AMR) is reducing treatment options for bacterial infections in animals and man. A number of antimicrobial drug classes are used in both human and veterinary medicines, *e*.*g*., *β*-lactams, aminoglycosides, fluoroquinolones, macrolides, sulfonamides, and tetracyclines [1]. In food animals such as beef cattle antimicrobial treatments are used to sustain animal health and welfare [2]. The treatments, however, result in the exposure of animal enteric bacteria to the drug or its active metabolites reaching the intestine. This may promote AMR in the enteric bacteria. Some of animal enteric bacteria are potential foodborne pathogens. Human food-borne infections with AMR bacterial strains are challenging to treat [1,3]. Furthermore, the ingested AMR strains not causing disease can become a part of the human enteric microbial community [4], and transfer genetic determinants of resistance to other human bacteria [5]. Prevention of this potential food-borne risk requires mitigation of AMR in enteric bacteria of food animals treated by antimicrobial drugs.

Pharmacodynamic models can be used to assess how antimicrobial drugs or their active metabolites that reach the lower intestine of treated animals affect the enteric bacteria in the anaerobic conditions of the intestine [6]. The models utilize measurements of bacterial susceptibility to the antimicrobials, such as the minimum inhibitory concentration (MIC) of the drugs [7–9]. Currently, the measurements obtained *in vitro* in aerobic conditions are utilized [10]. However, potential foodborne pathogens among the animal enteric bacteria such as Gram-negative bacilli *Escherichia coli* and *Salmonella enterica* are facultative anaerobes. Such bacteria experience changes in physiology and population growth rates in anaerobic conditions [11,12]. A number of bactericidal antimicrobial drug classes target growing-to-divide bacterial cells [13–17] and therefore the bactericidal activity depends on the bacterial population growth rates [18,19]. Other antimicrobial drug classes target bacterial functions that can be affected by the physiological changes that facultative anaerobe bacteria experience in anaerobic conditions [20–22]. Therefore, susceptibility of facultative anaerobe bacteria to different antimicrobial drug classes likely changes between aerobic and anaerobic conditions. The objective of this study was to investigate differences in the susceptibility, as measured by MIC, in aerobic *vs*. anaerobic conditions of generic *E*. *coli* and *Salmonella enterica* of diverse serovars to major antimicrobial drug classes: *β*-lactams, aminoglycosides, fluoroquinolones, classical macrolides, azalides, sulfonamides, and tetracyclines. Bacterial isolates originally obtained from cattle feces were used.

## Materials and Methods

### Bacterial isolates

Twenty five *E*. *coli* and 25 *Salmonella enterica* isolates were selected through systematic random sampling from existing isolate collections at the Department of Diagnostic Medicine/Pathobiology of Kansas State University. The isolates were obtained from cattle feces by earlier field studies in the U.S. beef feedlots. All available generic *E*. *coli* isolates were included in one sampling frame for the random selection of 25 isolates. A separate frame was implemented for the random selection of isolates from the collection of each available serovar of *Salmonella enterica* so as to select a total of 25 isolates of diverse serovars. After the antimicrobial susceptibility testing, all the isolates of *E*. *coli* and *Salmonella* which would be interpreted as susceptible to the tested drug based on the clinical breakpoint interpretation for human infection [7] were included in the statistical analyses (the isolates which would be interpreted as resistant were excluded.) This design ensured that *n*≥30 isolates would be available for the statistical analysis of the difference in the isolates’ susceptibility between the two conditions for each drug tested. The analyzed set of isolates contained *E*. *coli* of serovars O26, O45, O103, O111, O145, and *Salmonella enterica* of serovars Agona, Anatum, Give, Infantis, Kentucky, Montevideo, Muenchen, Oranienburg, and Typhimurium.

### Antimicrobials

For each bacterial isolate, its MIC value in anaerobic conditions and its MIC value in aerobic conditions were determined using E-Test^®^ as described below for the following drugs as representatives of their antimicrobial classes: ampicillin for older *β*-lactams aminopenicillins and ceftriaxone for newer *β*-lactams cephalosporins; gentamicin and kanamycin for aminoglycosides; enrofloxacin for fluoroquinolones; erythromycin for classic macrolides; azithromycin for azalides; sulfamethoxazole/trimethoprim for sulfonamides; and tetracycline for tetracyclines.

### Testing antimicrobial susceptibility of bacterial isolates

The E-Test^®^ (bioMerieux, Durham, NC, USA) assay was chosen to test antimicrobial susceptibility of the isolates for two reasons. Firstly, it is suitable for measuring MIC in both aerobic and anaerobic conditions [23–27]. Secondly, the assay evaluates the exact MIC value on continuous scale (rather than MIC ranges); this was particularly useful for comparing the MIC values for individual isolates between the two conditions. *Escherichia coli* ATCC 25922 strain was included in the experiments for each drug as a reference strain; the MIC for this strain in aerobic conditions was checked against the expected ranges, as per E-Test^®^ manufacturer's recommendations.

Each bacterial isolate used was purified at the time of original isolation and stored at -80°C. From the thawed freezer tube, the isolate was plated using an inoculation loop on a plate of the selected agar and incubated at 37°C for 24 hours. For the aerobic test, two to three colonies were picked from the agar plate using an inoculation loop and suspended in 9 mL Mueller-Hinton broth (MHB) (BD Diagnostic, Sparks, MD, USA) until the optical density of the suspension was equivalent to a 0.5 McFarland turbidity standard. A sterile cotton swab applicator was used to plate the suspension (approximately 100 μL, as per E-Test^®^ manufacturer’s recommendations) on a new plate of the selected agar. The E-Test^®^ strip containing the antimicrobial drug was placed in the center of this plate, and the plate was incubated upside down at 37°C for 24 hours, after which the result was read and recorded. For reading the result, an ellipse-shaped zone of bacterial growth inhibition centered along the strip was evaluated visually; the MIC value was read as the drug concentration on the strip where the ellipse’s edge intersected the strip, as per E-Test^®^ manufacturer's recommendations. For the anaerobic test, all these procedures were performed in an anaerobic chamber (Thermo Fisher Scientific Inc., Waltham, MA, USA); a sufficient number of colonies were picked up from the first agar plate for the optical density of the suspension in MHB to be equivalent to a 0.5 McFarland turbidity standard. With this experimental design, the bacterial culture was exposed to the aerobic or anaerobic conditions for 24 hours (while on the first agar plate) prior to the test of its antimicrobial susceptibility, and for further 24 hours during the test (while on the second agar plate with the test strip). The aerobic and anaerobic tests were performed separately on Mueller-Hinton (MH) agar (Becton, Dickinson and Company, Sparks, MD, USA) and on a more complex tryptic soy agar with 5% sheep blood (BAP) agar (Remel, Lenexa, KS, USA).

A satellite experiment was performed to investigate how quickly from the start of bacterial exposure to anaerobic conditions the physiological changes in the bacteria lead to a detectable change in the bacterial susceptibility to antimicrobials. The experiment was conducted with five isolates of *E*. *coli* and five isolates of *Salmonella enterica* selected at random from the isolates in the study interpreted as susceptible to all antimicrobials tested based on the clinical breakpoint interpretation for human infection [7]. The experiment was performed with the 10 isolates for ampicillin, gentamicin, kanamycin, and enrofloxacin, and with the five *E*. *coli* isolates only for azithromycin. The experiment with a given antimicrobial drug was performed on either MH or BAP agar. In the experiment, each of 10 isolates was plated in duplicate on two plates of the selected agar and incubated at 37°C for 24 hours aerobically. From the first plate, two to three colonies were picked to perform E-Test^®^ on a new plate aerobically. The second plate was transferred to the anaerobic chamber, and two to three colonies were picked up from the plate to perform E-Test^®^ anaerobically on a new plate. These tests provided measurements of the isolate’s susceptibility to the antimicrobials in aerobic conditions, and in anaerobic conditions following 0 hours of adaptation to these conditions. Each of the 10 isolates was also freshly plated in the anaerobic chamber on a third agar plate. Following each 12 hours of incubation in the chamber at 37°C, colonies were picked from this plate (sufficient number of colonies for the optical density of the suspension in MHB to be equivalent to a 0.5 McFarland turbidity standard) to perform E-Test^®^ anaerobically on a new plate. This provided measurements of the isolate’s susceptibility to the antimicrobials in anaerobic conditions following a 12, 24, 36, and 48 hour period of adaptation to these conditions.

### Statistical analysis

Statistical significance of the difference between the MIC values for the isolates in the anaerobic and aerobic tests was evaluated for each bacterial species, antimicrobial drug tested, and agar media combination. The statistical significance was evaluated using the *t*-test for paired samples assuming heteroscedasticity implemented in Microsoft Excel 2013^®^ software for Windows (Microsoft Corporation, Redmond, WA, USA); and using the nonparametric Wilcoxon signed-rank test implemented in SigmaPlot^TM^ v. 13.0 software (Systat Software Inc., San Jose, CA, USA). Descriptive statistics of the distributions of the MIC values and differences between the MIC values in the two conditions for the isolates were obtained in Microsoft Excel 2013^®^ software. Potential correlation between the relative magnitude of the MIC values of individual isolates in aerobic and anaerobic conditions across the isolates tested was evaluated using the nonparametric Spearman correlation coefficient implemented in SigmaPlot^TM^ v. 13.0 software. Figures were prepared in SigmaPlot^TM^ v. 13.0 software.

## Results

**Fig 1:**
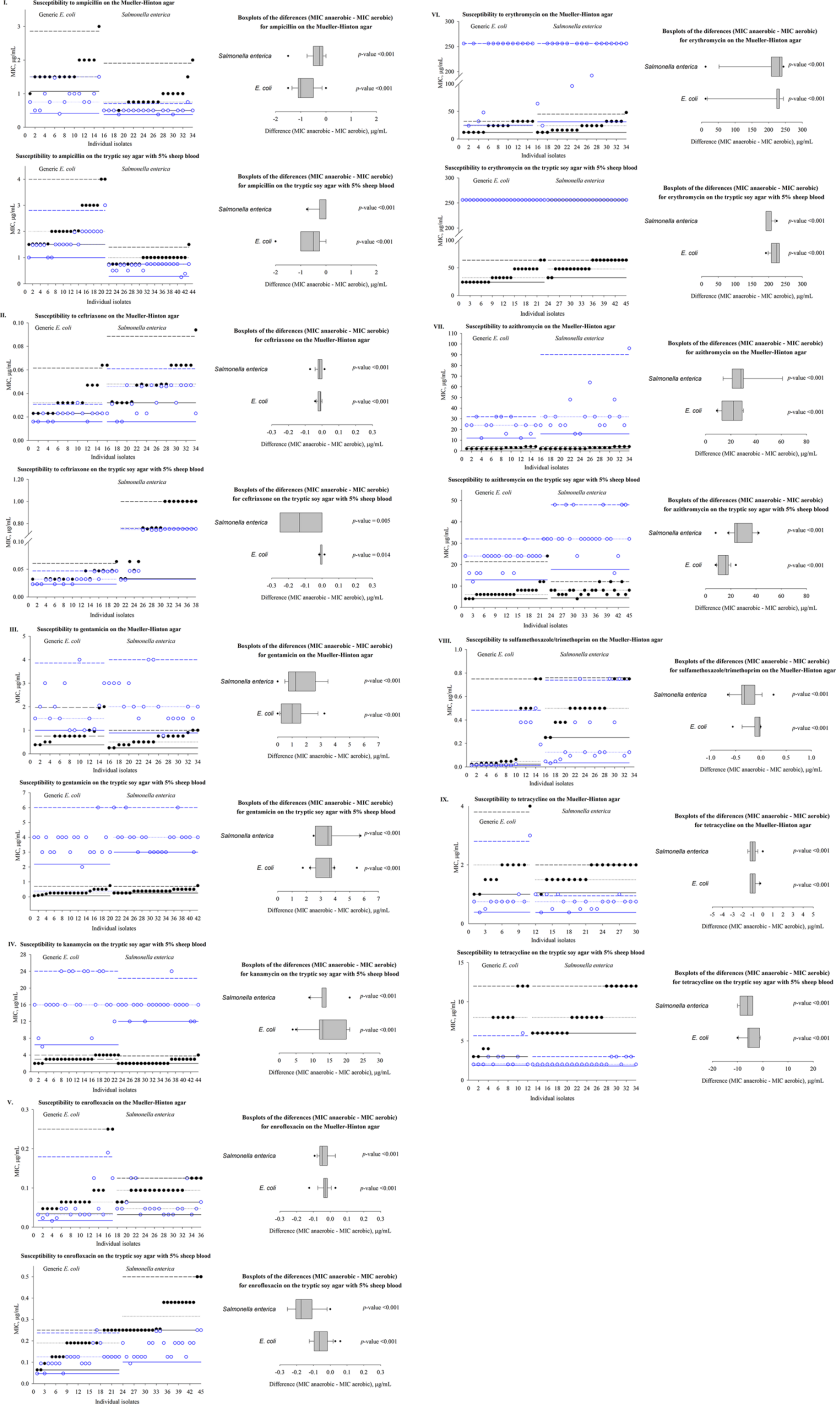
Antimicrobial susceptibility of *E*. *coli* and *Salmonella enterica* isolates of diverse serovars in aerobic and anaerobic conditions after a 24-hour adaptation to the conditions prior to the testing. Box-plots of the differences in susceptibility of the isolates between aerobic and anaerobic conditions (the *p*-value of the Wilcoxon signed rank test for each difference is included). Legend: I—Ampicillin. II—Ceftriaxone. III—Gentamicin. IV–Kanamycin. V–Enrofloxacin. VI–Erythromycin. VII–Azithromycin. VIII–Sulfamethoxazole/Trimethoprim. IX–Tetracycline. On the scatter plots, for each isolate, the solid black circle is the MIC in aerobic test and the hollow blue circle is the MIC in anaerobic test. The isolates are sorted in the order of increasing MIC values in aerobic conditions. For each bacterial species, the black lines denote the distribution of the MIC values across the isolates in aerobic conditions: solid line– 1% quartile, long dash–median, and short dash– 99% quartile. The magenda lines denote the distribution of the MIC values across the isolates of that bacterial species in anaerobic conditions: solid line– 1% quartile, long dash–median, and short dash– 99% quartile.

Descriptive statistics of the MIC value distributions for the antimicrobial drugs tested in aerobic and anaerobic conditions for the *E*. *coli* and *Salmonella enterica* isolates included in the statistical analyses are given in Table 1. The isolates (*n*≥30) included in the analyses were those that would be interpreted as susceptible to the antimicrobial drugs based on the MIC values in the aerobic tests and the clinical breakpoint interpretation for human infection [7]. The descriptive statistics of the differences between the MIC values in the anaerobic and aerobic tests, and the results of tests of statistical significance of the differences are also included in Table 1. The results are presented graphically in Fig 1.

**Table 1:** Descriptive statistics of the MIC distributions, and descriptive statistics and statistical significance of the differences between the MIC values in anaerobic and aerobic conditions for antimicrobials tested (E-Test^®^ on Mueller-Hinton agar) for *E*. *coli* and *Salmonella enterica* of diverse serovars.

Antimicrobial drug class	Representative drug used	Species	Number of isolates tested, *n*	MIC in aerobic tests, median (1%, 99% quartiles)	MIC in anaerobic tests after a 24-hour adaptation, median (1%, 99% quartiles)	Difference (MIC anaerobic-MIC aerobic), median (1%, 99% quartiles)	Paired *t*-test, *p*-value	Wilcoxon signed-rank test, exact *p*-value
Older *β*-lactams aminopenicillins	Ampicillin	*E*. *coli*	15	1.5 (1.1, 2.9)	0.75 (0.4, 1.5)	-1.0 (-1.5, -0.04)	<0.001	<0.001
		*Salmonella enterica*	19	0.7 (0.5, 1.9)	0.5 (0.4, 0.7)	-0.25 (0, 1.4)	<0.001	<0.001
Newer *β*-lactams third generation cephalosporins	Ceftriaxone	*E*. *coli*	15	0.03 (0.02, 0.06)	0.02 (0.02, 0.03)	-0.01 (-0.04, 0.00)	0.001	<0.001
		*Salmonella enterica*	19	0.05 (0.03, 0.09)	0.05 (0.02, 0.06)	-0.02 (-0.07, 0.01)	0.002	<0.001
Aminoglycosides	Gentamicin	*E*. *coli*	20	0.75 (0.4, 2.0)	1.5 (1.0, 3.9)	1.0 (0.04, 3.1)	0.001	<0.001
		*Salmonella enterica*	22	0.5 (0.25, 1)	2.0 (0.9, 4.0)	1.25 (0.1, 3.5)	<0.001	<0.001
Fluoroquinolones	Enrofloxacin	*E*. *coli*	17	0.06 (0.03, 0.25)	0.03 (0.02, 0.18)	-0.03 (-0.11, 0.03)	0.001	<0.001
		*Salmonella enterica*	19	0.09 (0.06, 0.13)	0.05 (0.03, 0.13)	-0.05 (-0.09, 0.03)	<0.001	<0.001
Classic macrolides	Erythromycin	*E*. *coli*	15	24.0 (12.0, 32.0)	256.0 (25.1, 256.0)	224.0 (13.1, 244.0)	<0.001	<0.001
		*Salmonella enterica*	19	24.0 (12.0, 45.1)	256.0 (31.2, 256.0)	232.0 (19.2, 243.3)	<0.001	<0.001
Azalides	Azithromycin	*E*. *coli*	15	2.0 (2.0, 4.0)	24.0 (12.0, 32.0)	22.0 (9.1, 30.0)	<0.001	<0.001
		*Salmonella enterica*	19	2.0 (2.0, 4.0)	32.0 (16.0, 90.2)	28.0 (14.0, 86.4)	<0.001	<0.001
Sulfonamides	Sulfamethoxazole/Trimethoprim	*E*. *coli*	15	0.05 (0.02, 0.75)	0.02 (0.01, 0.48)	-0.04 (-0.52, -0.01)	0.026	<0.001
		*Salmonella enterica*	18	0.50 (0.25, 0.75)	0.13 (0.03, 0.75)	-0.32 (-0.65, 0.21)	<0.001	<0.001
Tetracyclines	Tetracycline	*E*. *coli*	11	2 (1, 3.8)	0.75 (0.4, 2.8)	-0.9 (-1.3, 0.0)	<0.001	<0.001
		*Salmonella enterica*	19	1.5 (1, 2)	0.8 (0.4, 1.0)	-1.0 (-1.5, -0.1)	<0.001	<0.001

Susceptibility of *E*. *coli* and *Salmonella enterica* of diverse serovars (*n*≥30) isolated from cattle feces to all antimicrobial drug classes considered was statistically significantly different in anaerobic compared with aerobic conditions (Table 1, Fig 1). The direction of the difference in susceptibility between the two conditions was similar for the two bacterial species for each antimicrobial class. In particular, susceptibility to *β*-lactams such as aminopenicillins and third generation cephalosporins, fluoroquinolones, sulfanomides, and tetracyclines increased significantly in anaerobic compared with aerobic conditions, as was demonstrated by lower MIC values in anaerobic conditions (Fig 1 parts I, II, V, VIII, and IX). Susceptibility to aminoglycosides, classic macrolides, and azalides decreased significantly in anaerobic compared with aerobic conditions, as was demonstrated by higher MIC values in anaerobic conditions (Fig 1 parts III, IV, VI, and VII).

The direction of the difference in susceptibility between aerobic and anaerobic conditions for both bacterial species to every antimicrobial drug tested was consistent between MH and BAP agar media, but the range of the difference varied between the media in some cases. For example, the range of the difference was wider on BAP agar *for E*. *coli* susceptibility to enrofloxacin (Fig 1 part V), but on MH agar for *Salmonella enterica* susceptibility to azithromycin (Fig 1 part VII). The two media differ in their composition, with BAP being a more complex agar containing 5% sheep blood. Thus, the differences in the results between the two media suggested a possibility that the change in bacterial antimicrobial susceptibility from aerobic to anaerobic conditions may be affected further by the substrates available to support the bacterial population growth.

A positive statistically significant correlation was observed between the relative magnitude of the MIC values in aerobic and anaerobic conditions on MH agar for individual *E*. *coli* and *Salmonella enterica* isolates for *β*-lactam antimicrobials (ampicillin, ceftriaxone), fluoroquinolone enrofloxacin, a classic macrolide erythromycin, and a sulfanomide sulfamethoxazole/trimethoprim (Table 2). That is, for these antimicrobial classes there was a tendency for the relative magnitude of susceptibility of an isolate, compared to the other isolates, to remain consistent between the two conditions (Table 2; Fig 1 parts I, II, V, VI, and VIII). No statistically significant correlation was observed between the relative magnitude of the MIC values in aerobic and anaerobic conditions on MH agar for individual isolates for aminoglycoside gentamicin, azalide azithromycin, or tetracycline (Table 2; Fig 1 parts III, VII, and IX). That is, for these antimicrobial classes the relative magnitude of susceptibility of an isolate, compared to the other isolates, was not consistent between the two conditions. A similar pattern of the correlations of the relative magnitude of the MIC values of individual isolates between the conditions was observed on BAP agar. With the exception of susceptibility to azithromycin for which a statistically significant positive correlation coefficient of 0.45 (*p* = 0.002) between the MIC values in aerobic and anaerobic conditions was observed on BAP agar. The correlation for kanamycin was evaluated on BAP agar only, and was not statistically significant (correlation coefficient of 0.28, *p* = 0.059); this was similar to the results for gentamicin on MH agar. The ranges of the MIC values for the isolates analyzed are given in Table 1; the results of the analysis of the correlations are given in Table 2.

**Table 2:** Correlations between the relative magnitude of the MIC values of individual isolates of *E*. *coli* and *Salmonella enterica* of diverse serovars in aerobic and anaerobic conditions across the isolates tested (E-Test^®^ on Mueller-Hinton agar).

Antimicrobial drug class	Representative drug used	Isolates tested, *n*	Spearman correlation coefficient of MIC aerobic with MIC anaerobic for individual isolates	*p*-value
Older *β*-lactams aminopenicillins	Ampicillin	34	0.65	<0.001
Newer *β*-lactams third generation cephalosporins	Ceftriaxone	34	0.59	<0.001
Aminoglycosides	Gentamicin	34	-0.30	0.085
Fluoroquinolones	Enrofloxacin	36	0.59	<0.001
Classic macrolides	Erythromycin	34	0.55	<0.001
Azalides	Azithromycin	34	0.10	0.589
Sulfonamides	Sulfamethoxazole/Trimethoprim	33	0.85	<0.001
Tetracyclines	Tetracycline	30	0.30	0.103

**Fig 2:**
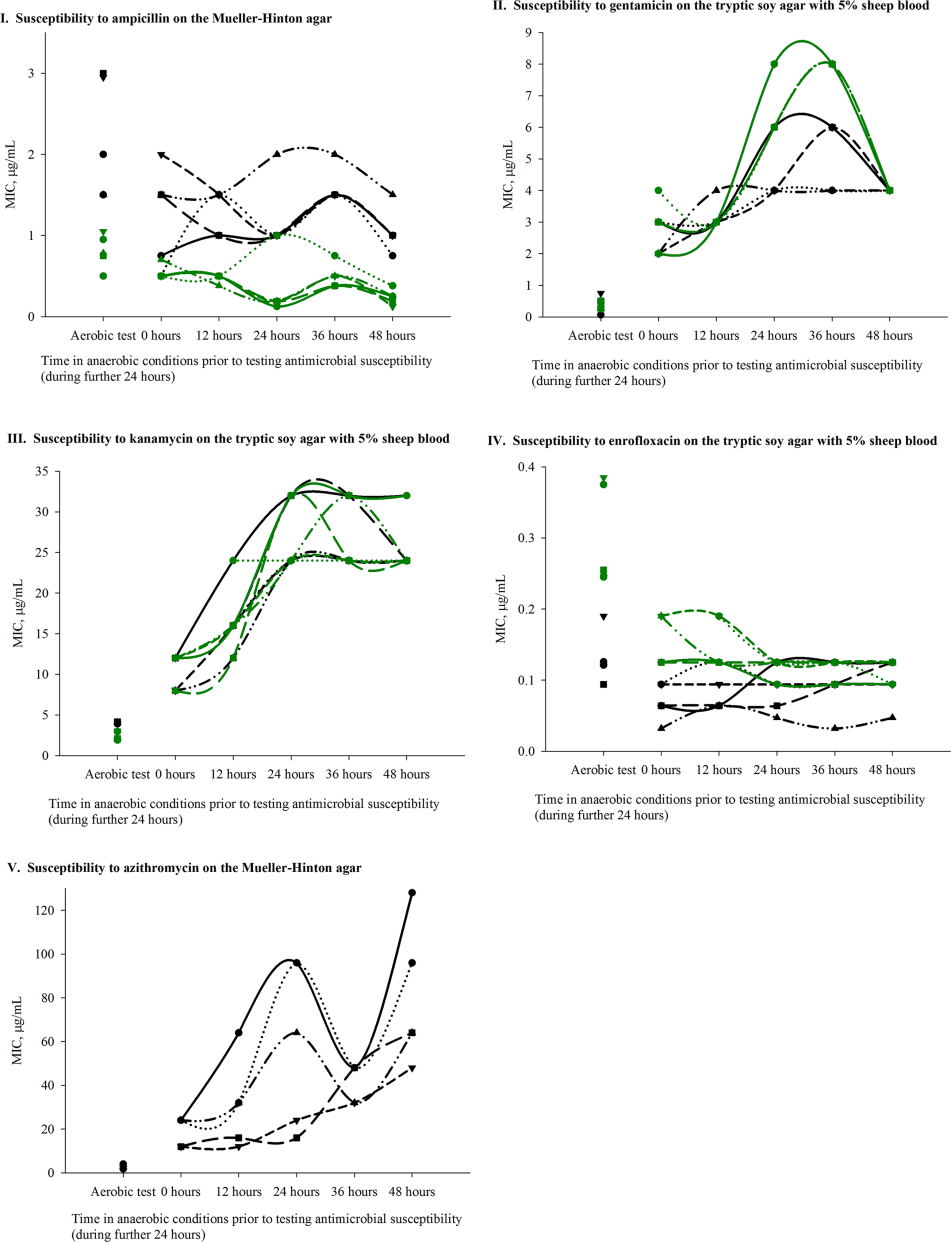
Dynamics of changes in antimicrobial susceptibility of individual *E*. *coli* (black symbols and lines) and *Salmonella enterica* (green symbols and lines) isolates of diverse serovars depending on the time of exposure to anaerobic conditions prior to testing the susceptibility in these conditions. Each isolate is represented by the same symbol in the scatter plot of the susceptibility in aerobic conditions, and the scatter and line plot of the susceptibility in anaerobic conditions. Legend: I–Ampicillin. II–Gentamicin. III–Kanamycin. IV–Enrofloxacin. V–Azithromycin.

In the satellite experiment, bacterial antimicrobial susceptibility in anaerobic conditions was evaluated following from 0 to 48 hours of exposure to these conditions. The results of this experiment suggested that adaptation of Gram-negative facultative anaerobic bacilli to anaerobic conditions is associated with dynamic changes in their antimicrobial susceptibility (Fig 2). Distinct patterns of the dynamics of the changes were observed for individual antimicrobial classes (Fig 2). Furthermore, although the overall direction of change in susceptibility (increase or decrease compared to aerobic conditions) for a given antimicrobial was similar among individual isolates of a given bacterial species, there was between-isolate variability in the dynamics or magnitude of the changes with time in anaerobic conditions (Fig 2).

## Discussion

The results of this study are consistent with the hypothesis that facultative anaerobe Gram-negative enteric bacteria can have different susceptibility to antimicrobials when exposed in aerobic *vs*. anaerobic conditions. Isolates of diverse serovars of *E*. *coli* (O26, O45, O103, O111, O145), and *Salmonella enterica* (Agona, Anatum, Give, Infantis, Kentucky, Montevideo, Muenchen, Oranienburg, Typhimurium) exhibited changes in their susceptibility to antimicrobial drugs (*n*≥30 isolates per drug) from aerobic to anaerobic conditions of the bacterial population growth. Susceptibility to *β*-lactams, including aminopenicillins and third generation cephalosporins, as well as to fluoroquinolones, sulfanomides, and tetracyclines increased significantly. Susceptibility to aminoglycosides, classic macrolides, and azalides decreased significantly. The isolates used had been obtained from cattle feces by earlier field studies in beef feedlots. In our view, the observed differences in bacterial antimicrobial susceptibility between the aeration conditions are likely independent of the isolate source. This premise, however, can be tested in the future by investigating susceptibility of isolates from other sources (*e*.*g*. from humans, monogastric animals, poultry or food products).

The observed differences in antimicrobial susceptibility from aerobic to anaerobic conditions of facultative anaerobe Gram-negative enteric bacteria are likely associated with physiological changes experienced by the bacteria. We conjuncture that the most influential may be alterations in the bacterial population growth rate, respiration, or metabolism, as well as changes in the uptake or activity of the antimicrobials due to the aeration conditions. This observational study did not aim to distinguish the contributing processes. We conjecture that the altered population growth rates likely affect the bacterial susceptibility to antimicrobial drugs that inhibit synthesis of the cell wall of vegetative, growing-to-divide bacteria, such as *β*-lactams. The altered metabolism can affect the susceptibility to antimicrobials that target major metabolic cell functions, such as macrolides, sulfonamides, and tetracyclines. The differences in activity of aminoglycosides in anaerobic conditions are better understood [20,28,29], and are associated with a reduced aminoglycoside uptake by the bacteria due to changes in bacterial respiration in anaerobic conditions [30].

This study was limited to *E*. *coli* and *Salmonella enterica* that were highly susceptible to antimicrobial drugs tested (*i*.*e*., the isolates would be interpreted as susceptible to the drugs based on the clinical breakpoint interpretation for human infection [7]). Such isolates were available for the study in sufficient quantities to allow meaningful statistical analyses of the changes in the bacterial susceptibility to the antimicrobials between aerobic and anaerobic conditions. By their virtue, the isolates had a range of relatively low MIC values for the antimicrobials (Table 1). It is possible that the magnitude of the changes in susceptibility between the conditions can differ for less susceptible isolates that manifest relatively high MIC values (*i*.*e*., isolates that would be interpreted as intermediate or resistant to the antimicrobials based on the clinical breakpoint interpretation), though the direction of the change can remain the same. Further, the correlation between the relative magnitude of the susceptibility in aerobic and anaerobic conditions may also differ for less susceptible isolates. Within the MIC value range of the susceptible isolates analyzed, there were positive and statistically significant correlations between the relative magnitude of susceptibility of individual isolates in aerobic and anaerobic conditions for *β*-lactams, fluoroquinolones, classic macrolides and sulfanomides, but not for aminoglycosides, azalides or tetracyclines. It was not feasible to obtain a sufficiently large set of the isolates with more diverse susceptibility levels (more variable MIC values for the antimicrobials tested) at the time of this study.

At least one drug from each of the antimicrobial drug classes considered in this study is labelled for use to treat cattle in the U.S. Pharmacodynamic models can be developed to describe how the drugs or their active metabolites that reach the intestines of the treated animals affect the enteric bacterial populations and their antimicrobial resistance. The models require measurements of the antimicrobial susceptibility of the bacteria in the conditions of the exposure. The exposure likely occurs in the lower intestine where the conditions are anaerobic. The results of this study demonstrate that susceptibility of Gram-negative facultative anaerobe enteric bacteria, in particular important foodborne pathogens *E*. *coli* and *Salmonella enterica*, changes in anaerobic conditions. Moreover, the change may depend on the time in anaerobic conditions. Therefore, pharmacodynamic models aiming to evaluate the impact of antimicrobial use on antimicrobial resistance in enteric bacteria of the treated host should utilize measurements of bacterial susceptibility that are obtained anaerobically and reflect the time bacteria spend in anaerobic conditions of the host’s gastrointestinal tract prior to the antimicrobial exposure.
